# A *Dalbergia odorifera* extract improves the survival of endotoxemia model mice by inhibiting HMGB1 release

**DOI:** 10.1186/s12906-017-1725-0

**Published:** 2017-04-12

**Authors:** Hyuk Soo Choi, Jin-A Park, Jung Seok Hwang, Sun Ah Ham, Taesik Yoo, Won Jin Lee, Kyung Shin Paek, Ho-Chul Shin, Chi-Ho Lee, Han Geuk Seo

**Affiliations:** 1 College of Animal Bioscience & Technology, Konkuk University, 120 Neungdong-ro, Gwangjin-gu, Seoul 05029 Korea; 2 Department of Veternary Pharmacology and Toxicology, College of Veterinary Medicine, Konkuk University, 120 Neungdong-ro, Gwangjin-gu, Seoul 05029 Korea; 3 Department of Nursing, Semyung University, Semyung-ro, Jechon, Chungbuk 390-711 Korea

**Keywords:** *Dalbergia Odorifera*, Endotoxemia, HMGB1, Inflammation, Nitric oxide

## Abstract

**Background:**

*Dalbergia odorifera* T. Chen (Leguminosae) is an indigenous medicinal herb that is widely used as a popular remedy in northern and eastern Asia. However, the cellular mechanisms underlying the biological activity of *D. odorifera* are not fully elucidated.

**Methods:**

Anti-inflammatory effect of *D. odorifera* extract (DOE) was determined through intraperitoneal injection in a mouse model of endotoxemia induced by lipopolysaccharide (LPS). RAW 264.7 cells, a murine macrophage, were also treated with LPS to generate a cellular model of inflammation, and investigated the anti-inflammatory activity and underlying mechanisms of DOE and its constituent isoliquiritigenin.

**Results:**

DOE dose-dependently inhibited LPS-induced release of high mobility group box 1 (HMGB1), a late proinflammatory cytokine, and decreased cytosolic translocation of HMGB1 in RAW264.7 cells. This inhibitory effect of DOE on HMGB1 release was observed in cells treated with DOE before or after LPS treatment, suggesting that DOE is effective for both treatment and prevention. In addition, DOE significantly inhibited LPS-induced formation of nitric oxide (NO) and expression of inducible NO synthase (iNOS) in a dose-dependent manner. These effects of DOE were accompanied by suppression of HMGB1 release triggered by LPS, suggesting a possible mechanism by which DOE modulates HMGB1 release through NO signaling.

Isoriquiritigenin, a constituent of DOE, also attenuated LPS-triggered NO formation and HMGB1 release in RAW264.7 cells, indicating that isoriquiritigenin is an indexing molecule for the anti-inflammatory properties of DOE. Furthermore, c-Jun N-terminal kinase, but not extracellular signal-regulated kinase and p38, mediated DOE-dependent inhibition of HMGB1 release and NO/iNOS induction in RAW 264.7 cells exposed to LPS. Notably, administration of DOE ameliorated survival rates in a mouse model of endotoxemia induced by LPS, where decreased level of circulating HMGB1 was observed.

**Conclusion:**

These results suggest that DOE confers resistance to LPS-triggered inflammation through NO-mediated inhibitory effects on HMGB1 release.

## Background

High mobility group box 1 (HMGB1) is a evolutionarily conserved non-histone nuclear protein that is ubiquitously expressed in most eukaryotic cells [1]. In the nucleus, HMGB1 interacts with histones to regulate key processes including replication, transcription, recombination, genome stabilization, and repair [2]. In addition to its nuclear localization, HMGB1 is secreted into the extracellular compartment by immunocompetent cells activated upon exposure to pathogen-associated molecular patterns such as lipopolysaccharide (LPS), in which it acts as a proinflammatory cytokine [3]. The proinflammatory activity of HMGB1 was first demonstrated in a sepsis mouse model as a late mediator of lethality, in which HMGB1 is actively secreted by activated macrophages [4]. In addition, serum levels of HMGB1 were elevated with delayed kinetics in sepsis patients showing overwhelmed cellular inflammatory and immune responses, resulting in multiple organ failure, tissue damage, and death [4–6]. Recent studies indicated the roles of extracellular HMGB1 in inflammation-associated disease pathogenesis; the administration of neutralizing anti-HMGB1 antibodies or HMGB1 inhibitors significantly reduces inflammatory conditions in sepsis, arthritis, colitis, and ischemic reperfusion [4, 7–9]. Notably, blockade of HMGB1 signals by these reagents confers cellular protection from delayed endotoxin lethality in a mouse model of endotoxemia, even when applied after the acute phase of cytokine responses has peaked, indicating that HMGB1 is a novel target for inflammatory diseases [4, 10, 11]. As part of the endeavor to target HMGB1 release, we demonstrated that rosiglitazone, an activator of the nuclear protein peroxisome proliferator-activated receptor (PPAR) γ, inhibits LPS-triggered release of HMGB1 in RAW264.7 cells [12]. Furthermore, PPARγ-mediated upregulation of SIRT1 expression participates in the inhibitory action of PPARγ through deacetylation-mediated interaction of SIRT1 and HMGB1, leading to improvement of the survival of endotoxemia model mice [13, 14]. Based on the proinflammatory properties of HMGB1, the blockade of its release into the extracellular milieu is a promising molecular target for the diseases associated with inflammatory disorders.

Herbal extracts and components from *Prunella vugaris*, Danggi, and mung bean have recently been demonstrated to effectively inhibit HMGB1 release induced by LPS [15–17]. Several molecular mechanisms by which herbal components effectively block HMGB1 release are postulated [18]. Glycyrrhizin inhibits LPS-triggered HMGB1 release through binding to HMGB1 in order to induce conformational changes that prevent DNA binding [18, 19]. Furthermore, epigallocatechin-3-gallate and tanshinone IIA stimulate autophagic degradation and endocytic uptake of HMGB1 in RAW264.7 cells, respectively [20, 21]. On the other hand, *Dalbergia odorifera*, a folk medicinal herb, exhibits a beneficial effect in inflammatory conditions via the induction of heme oxygenase (HO)-1, an anti-inflammatory protein, in murine RAW 264.7 macrophages and BV2 microglial cells [22, 23]. These anti-inflammatory activities of *D. odorifera* stem from a number of compounds such as flavonoids, phenolic constituents, and quinines isolated from its heartwood [24, 25]. Given the ability of several herbal components to inhibit LPS-triggered cellular responses for inflammation, we investigated the effect of a 60% ethanol extract of *D. odorifera* on HMGB1 release in LPS-treated RAW264.7 cells and a mouse model of endotoxemia. Here, we report that a *D. odorifera* extract (DOE) inhibited LPS-triggered release of HMGB1 in macrophages and an animal model of endotoxemia. DOE suppressed LPS-induced nitric oxide (NO) signaling by preventing the c-Jun N-terminal kinase (JNK) signaling cascade, leading to blockade of HMGB1 release into the extracellular milieu.

## Methods

### Materials

LPS (*Escherichia coli* 0111:B4), Ponceau S solution, *N* ^G^-monomethyl-L-arginine (L-NMMA), daidzein, formononetin, isoliquilirigenin, and a rabbit polyclonal antibody specific for β-actin were obtained from Sigma-Aldrich Co. (St. Louis. MO, USA). Dalbergin were purchased from R&D chemicals (Bethesda, MD, USA) and the sativanone standard was generously provided by Dr. Hiroshi Noguchi (School of Pharmaceutical Sciences, University of Shizuoka, Shizuoka, Japan). Piperidinewas purchased from JUNSEI (Tokyo, Japan). SP600125 [Anthra (1,9-cd)pyrazol-6(2H)-one] was obtained from Calbiochem (La Jolla, CA, USA). Monoclonal antibodies specific for inducible NO synthase (iNOS) and HMGB1 were supplied by Epitomics (Burlingame, CA, USA). Rabbit polyclonal antibodies specific for p38, phospho-p38, extracellular signal-regulated kinase (ERK), phospho-ERK, JNK, and phospho-JNK were purchased from Cell Signaling (Beverly, MA, USA). Monoclonal antibodies specific for lamin B and α-tubulin were purchased from Santa Cruz Biotechnology (Dallas, TX, USA).

### Preparation of DOE

DOE was prepared according to a previously described method [26]. Briefly, heartwood of *D. odorifera* was purchased from a Korean medicinal herb store in Seoul in May 2012. An authenticated voucher specimen (KULBM-1205) was deposited in the Herbarium at the College of Animal Bioscience and Technology, Konkuk University (Seoul, Korea). Dried heartwood of *D. odorifera* (100 g) was extracted three times with 60% ethanol under rotation of 120 rpm for 1 h. After evaporation of the solvent under a vacuum, the extract (10 g) was reconstituted in dimethyl sulfoxide to a concentration of 10 mg/ml and then stored at −20 °C until use.

### LC-MS/MS analysis

LC/MS/MS analysis was performed using an Agilent series 1100 HPLC (Agilent Technologies, CA, USA) instrument, equipped with a G1311A quart pump, a G1313A autosampler, a G1322A degasser, a G1316A column oven, and an API 3200™ MS/MS system (Applied Biosystems, NY, USA). Chromatographic separation was performed on a Waters XBridge™ C18 analytical column (2.0 × 100 mm, 3.5 μm particle size, Milford, USA). The binary solvent system consisted of a mixture of: (A) 0.1% acetic acid in water, and (B) 0.1% formic acid in acetonitrile. The sample (10 μL) was then injected at a flow rate of 0.2 mL/min under the following gradient elution program: (i) 0–3 min, 95% A; (ii) 3–5 min, 95 to 5% A; (iii) 5–10 min, 5% A; (iv) 10–13 min, 5 to 95% A; and (v) 13-17 min, 95% A. All multiple reaction monitoring (MRM) transitions of the analytes and other experimental parameters are compiled in Table 1. Analyst 1.4.2 software (ABI) was used for data management and control. The ion spray voltage (IS) and capillary temperature were set at 5.5 kV and 350 °C, respectively. Nitrogen was used as a collision gas with ion source gas 1 (GS1) and ion source gas 2 (GS2) at 40 psi and 60 psi, respectively.

**Table 1:** LC-MS/MS parameters for quantitation and confirmation

*Analyte*	*Retentiontime (min)*	*Q1 mass(m/z)*	*Q3 mass(m/z)*	*Declustering potential* *DP(V)*	*Entrance potential* *EP(V)*	*Collision energy* *CE(V)*	*Collision cell exit potential CXP(V)*
Isoliquiritigenin	10.03	257.212	137.200	66	10.5	27	4
			147.200	66	10.5	25	4
Daidzein	9.67	255.157	91.200	86	11	49	4
			65.100	86	11	71	4
Formononetin	10.13	269.215	197.200	186	12	45	4
			89.200	186	12	93	4
Dalbergin	10.12	269.164	152.000	111	10.5	61	4
			254.000	111	10.5	29	10
Piperidine	1.27	86.175	69.100	41	7.5	19	4
			41.000	41	7.5	29	4
Sativanon	10.21	301.262	223.000	56	8.5	15	4
			102.400	56	8.5	43	4

### Cell culture

Murine RAW 264.7 macrophages were incubated in DMEM containing 10% heat-inactivated fetal calf serum and 1% antibiotics at 37 °C under a condition of 5% CO_2_ and 95% air.

### Western blot analysis

Aliquots of total cell lysates were subjected to immunoblot analysis as essentially described [14]. In brief, whole-cell lysates containing 30 μg protein per lane were fractionated on a 10% SDS-polyacrylamide gel electrophoresis (PAGE) and blotted onto a polyvinylidene difluoride (PVDF) membrane (Amersham Biosciences UK Ltd., UK; Hybond-P^+^, 0.45 μm pore). Following blocking with 5% nonfat milk, the membranes were probed with specific antibodies. Finally, after reaction with a peroxidase-conjugated goat antibody, immuno-reactive proteins were visualized by West-ZOL Plus (iNtRON Biotechnology).

### Determination of secreted HMGB1

HMGB1 secretion was determined according to an essentially described method [14]. Briefly, the relative amounts of HMGB1 secreted into culture media were determined from conditioned culture media of equal numbers of cells. Equal volumes of conditioned culture media and 80% cold acetone were mixed and incubated at −20 °C for 1 h. Following centrifugation at 16,000×g for 10 min at 4 °C, the precipitates were washed with 80% cold acetone. The resulting pellets resuspended in SDS-PAGE sample buffer were subjected to Western blot analysis. As a loading control, Ponceau S staining was used.

### Measurement of NO

NO formation was measured spectrophotometrically as its stable oxidative metabolite, nitrite, formed by reactions with Griess reagents as described previously [27]. Briefly, 100 μl of culture medium was mixed with the same volume of Griess reagents [one part of 1% sulfanilamide in 0.1 M HCl and one part of 0.1% N-(1-naphthyl)ethylenediamine dihydrochloride]. Following reaction for 10 min, optical density was determined at 550 nm in a microplate reader (Bio-Rad, Hercules, CA, USA). The nitrite concentration was determined with a curve calibrated using sodium nitrite standards. The background levels of nitrite in the culture media were subtracted from each sample.

### Fractionation of cytoplasmic and nuclear proteins

Cytosolic and nuclear fractions were prepared according to a previously described method [14]. Briefly, RAW264.7 cells washed with ice-cold PBS were suspended in lysis buffer (0.1 mM EDTA, 1 mM DTT, 10 mM KCl, 10 mM HEPES, and protease inhibitors, pH 7.9) and allowed to swell on ice for 15 min. After adding Nonidet P-40 (0.1%, final concentration), cell lysates were vortexed vigorously for 10 s. The cytosolic fraction, a supernatant, was obtained by centrifugation at 13,000×g for 30 s. The resulting pellet was washed with lysis buffer and resuspended in PRO-PREP Protein Extraction Solution (iNtRON Biotechnology). Following incubation for 30 min on ice, the supernatant, as the nuclear fraction, was collected by centrifugation at 13,000×g at 4 °C for 15 min. Bradford method was used to determine protein concentration.

### Animal model of endotoxemia and survival test

The Institutional Animal Care and Use Committee of Konkuk University approved all animal studies of the present study (approval number: KU15140). Endotoxemia was induced by infusion of bacterial endotoxin by intraperitoneal injection (10 mg/kg, *E. coli* LPS 0111:B4) into BALB/c mice (male, 6-week-old, 20–25 g) as described previously [14]. Briefly, BALB/c mice purchased from Koatech (Pyeongtaek, Korea) were housed in a pathogen-free environment. Mice were randomly assigned to the following groups: injection of LPS (10 mg/kg), simultaneous injection of LPS (10 mg/kg) plus DOE (20 mg/kg), and DOE (20 mg/kg) administration after LPS (10 mg/kg) infusion. Follow LPS injection, survival rate was assessed for up to 2 weeks to confirm the additional late deaths. To measure plasma HMGB1 levels, sepsis was induced in mice by LPS injection in the absence or presence of DOE as described above. After 20 h, blood was collected and allowed to clot at room temperature. Following centrifugation at 1600×g for 20 min, the HMGB1 levels in serum were detected by immunoblot.

### Statistical analysis

One-way ANOVA followed by the Tukey-Kramer test was used to determine the statistical significance.

## Results

### DOE inhibits LPS-triggered release of HMGB1 in RAW 264.7 cells

**Fig. 1:**
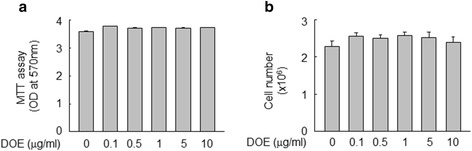
Cell viability was not affected by the concentrations of DOE tested. RAW 264.7 cells were treated with the indicated concentrations of DOE. After incubation for 24 h, cell viability was assessed by the MTT assay (**a**) or the trypan blue exclusion method (**b**). The results are plotted as the means ± S.E. (*n* = 4)

**Fig. 2:**
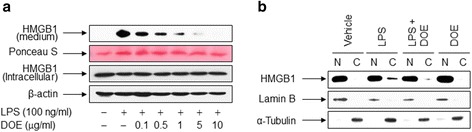
DOE inhibits LPS-triggered release of HMGB1 in RAW 264.7 cells. **a** Cells cultured for 24 h in serum-free medium were triggered by vehicle (DMSO) or LPS in the presence or absence of DOE. Following incubation for 24 h, aliquots of conditioned media or whole-cell lysates were analyzed by immunoblot analysis. Ponceau S staining and β-actin were used as the loading controls. **b** Cells were treated with vehicle (DMSO) or LPS in the presence or absence of DOE for 24 h, and then whole-cell lysates were fractionated into cytosolic (C) and nuclear (N) fractions. The localization of HMGB1 was analyzed by immunoblotting with the indicated antibodies. Representative blots from three independent experiments are shown

Cell viability was determined to choose the optimal dose ranges of DOE in RAW 264.7 cells. The cytotoxicity of DOE was not shown up to 10 μg/ml concentration (Fig. 1). Next, we investigated whether DOE affects cellular responses in inflammatory conditions, we examined the effect of DOE on LPS-triggered release of HMGB1 in murine RAW 264.7 macrophages. The HMGB1 release was increased in cells stimulated with LPS, whereas this increase was reduced in the presence of DOE in a concentration-dependent manner (Fig. 2a). In contrast with the levels of released HMGB1, neither LPS nor DOE affected the level of HMGB1 expression. These results suggest that DOE is associated with LPS-triggered HMGB1 release, but not HMGB1 expression.

Because HMGB1 translocates from the nucleus to the cytoplasm upon the activation of macrophages by inflammatory signals such as LPS [28], we examined whether DOE affects the translocation of HMGB1 to the cytoplasm induced by LPS. When RAW 264.7 cells were triggered by LPS, the re-localization of HMGB1 from the nucleus into the cytoplasm was increased, whereas this cytosolic translocation of HMGB1 was markedly inhibited in the presence of DOE (Fig. 2b). This indicates that DOE regulates HMGB1 release by inhibiting re-localization of HMGB1 into the cytoplasm in RAW 264.7 cells exposed to LPS.

### DOE attenuates LPS-triggered release of HMGB1 in RAW 264.7 cells even when administered after LPS treatment

**Fig. 3:**
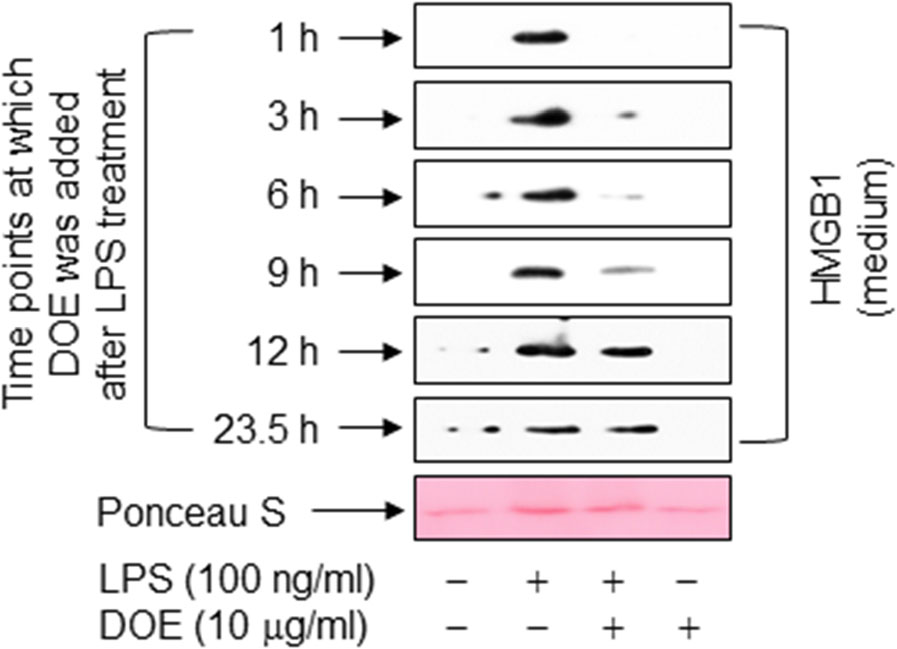
Treatment with DOE after LPS exposure attenuates LPS-triggered release of HMGB1 in RAW 264.7 cells. Cells cultured for 24 h in serum-free medium were triggered by vehicle (DMSO) or LPS. DOE was added at the indicated time points after LPS treatment for 24 h. Equal volumes of conditioned medium were subjected to immunoblot analysis to determine the levels of HMGB1. As a loading control, Ponceau S staining was used. The results are representative of three or four independent experiments

Because simultaneous treatment of cells with LPS and DOE effectively inhibited the release of HMGB1 induced by LPS, we investigated the effect of DOE supplied at various time points after LPS treatment. When cells were exposed to LPS, the level of HMGB1 was increased in the conditioned culture media at 24 h, and this enhancement was markedly attenuated by adding DOE to the cells after LPS treatment. This effect was observed in cells treated with DOE up to 9 h post-LPS treatment and, to a lesser extent, in cells treated with DOE up to 12 h post-LPS treatment (Fig. 3), indicating that DOE is useful for the prevention, as well as the treatment, of HMGB1 release.

### DOE inhibits LPS-induced NO formation in RAW 264.7 cells

**Fig. 4:**
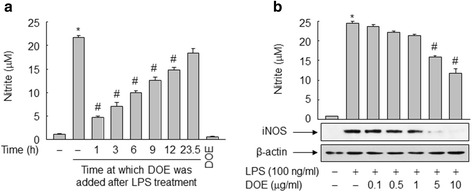
DOE inhibits LPS-triggered iNOS expression and NO formation in RAW 264.7 cells. **a** Cells cultured for 24 h in serum-free medium were triggered by vehicle (DMSO) or LPS. DOE was added at the indicated time points after LPS treatment for 24 h. The levels of nitrite were determined by incubation with Griess reagents. **b** Cells cultured for 24 h in serum-free medium were triggered by vehicle (DMSO) or LPS with or without indicated concentrations of DOE. After incubation for 24 h, aliquots of whole-cell lysates or conditioned media were subjected to immunoblot or nitrite analysis, respectively. The results are plotted as the means ± S.E. (*n* = 6). ^*^, *p* < 0.01 vs untreated group; ^#^, *p* < 0.01 vs LPS-treated group

NO was reported to mediate HMGB1 release in RAW 264.7 cells stimulated with LPS [29]; therefore, the effect of DOE on LPS-triggered NO formation was examined. The level of NO was markedly increased in LPS-stimulated RAW 264.7 cells for 24 h, whereas this increase was significantly reduced by adding DOE to the cells after LPS treatment. The significant inhibitory effect of DOE on the NO level was sustained up to 12 h post-LPS treatment (Fig. 4a). In accordance with these findings, DOE inhibited the expression of iNOS induced by LPS in a concentration-dependent manner, corroborating the effects of DOE observed on NO formation induced by LPS (Fig. 4b).

### DOE inhibits LPS-triggered release of HMGB1 through NO in RAW 264.7 cells

**Fig. 5:**
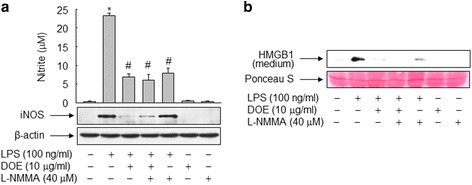
DOE inhibits LPS-triggered release of HMGB1 through inhibition of NO formation in RAW 264.7 cells. **a** and **b** Cells cultured for 24 h in serum-free medium were pretreated with or without L-NMMA and then stimulated with vehicle (DMSO) or LPS in the presence or absence of DOE. After incubation for 24 h, aliquots of whole-cell lysates or conditioned media were subjected to immunoblot or nitrite analysis, respectively (s). The levels of HMGB1 were analyzed by immunoblot using equal volumes of conditioned media (**b**). Ponceau S staining and β-actin were used as the loading controls. The blots are representative of three independent experiments. The results are plotted as the means ± S.E. (*n* = 6). ^*^, *p* < 0.01 vs untreated group; ^#^, *p* < 0.01 vs LPS-treated group

Because DOE effectively inhibited LPS-triggered iNOS expression and NO formation in RAW 264.7 cells, we examined the effect of DOE on HMGB1 release in the presence or absence of an iNOS inhibitor. Cells exposed to LPS exhibited marked increases in the NO level and iNOS expression, relative to unexposed control cells. These increases, however, were significantly suppressed in the presence of DOE. Furthermore, pretreatment with L-NMMA, a specific inhibitor of iNOS, caused a similar reduction in LPS-induced NO formation as that achieved with DOE. However, the results obtained upon combined treatment with both DOE and L-NMMA were not different from those obtained upon treatment with either alone (Fig. 5a).

To verify the involvement of NO signaling in the LPS-triggered release of HMGB1 in RAW 264.7 cells, we next examined the effects of L-NMMA on the LPS-triggered release of HMGB1 in the presence or absence of DOE. In LPS-treated RAW 264.7 cells, addition of L-NMMA inhibited HMGB1 release to a similar extent as DOE (Fig. 5b). However, the results obtained upon treatment with both DOE and L-NMMA were not different from those obtained upon treatment with either alone, indicating that the inhibitory activity of DOE is mediated through the NO signaling pathway (Fig. 5b).

### Identification of DOE constituents

**Fig. 6:**
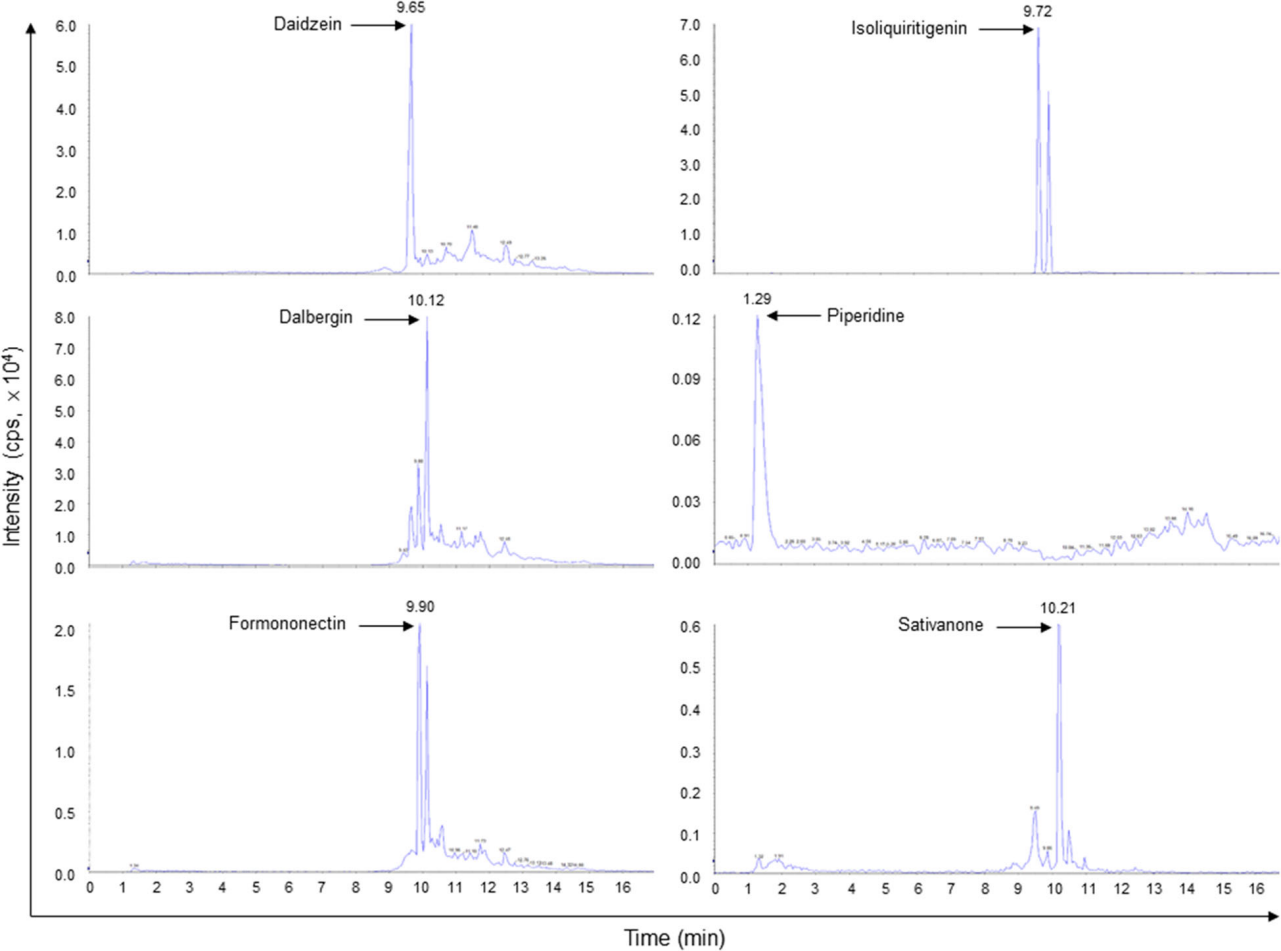
LC-MS/MS analysis. Chromatographic separation was performed on a C18 analytical column eluted with an acetonitrile gradient at 0.2 ml/min. Isoliquiritigenin, daidzein, formononetin, dalbergin, piperidine, and sativanone were identified using authentic standards

Since the bioactive phenolic compounds were isolated from the *D. odorifera* [25], we analyzed the constituents of DOE by LC-MS/MS. Representitive LC-MS/MS profiles of DOE were shown, and six phenolic components were identified as isoliquiritigenin, daidzein, formononetin, dalbergin, piperidine, and sativanone by comparison with authentic standards (Fig. 6).

### Anti-inflammatory effect of isoliquiritigenin

**Fig. 7:**
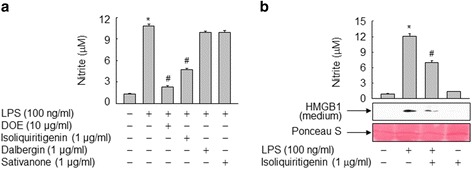
Isoliquiritigenin inhibits LPS-induced NO formation and HMGB1 release in RAW264.7 cells. **a** Cells cultured for 24 h in serum-free medium were stimulated with vehicle (DMSO) or LPS in the presence of DOE, isoliquiritigenin, dalbergin, or sativanone. After incubation for 24 h, aliquots of conditioned media were subjected to nitrite analysis. **b** Cells cultured for 24 h in serum-free medium were triggered by vehicle (DMSO) or LPS in the absence or presence of isoliquiritigenin. After incubation for 24 h, equal volumes of conditioned medium were subjected to nitrite analysis or immunoblot analysis to determine the levels of HMGB1. Ponceau S staining was used as a loading control. The results are plotted as the means ± S.E. (*n* = 6). ^*^, *p* < 0.01 vs untreated group; ^#^, *p* < 0.01 vs LPS-treated group

Based on the published reports regarding the anti-inflammatory activity of DOE constituents [30–32], we examined whether some component of DOE identified in the present study could affect NO formation in RAW264.7 cells exposed to LPS. In line with previous report [30], isoliquiritigenin, but not dalbergin and sativanone, significantly inhibited LPS-induced NO formation in RAW264.7 cells (Fig. 7a). In addition, isoliquiritigenin also inhibited the HMGB1 release in RAW264.7 cells treated with LPS, suggesting that the inhibitory effect of DOE through the NO signaling pathway on HMGB1 release can be mainly attributed to the isoliquiritigenin component (Fig. 7b).

### DOE inhibits LPS-triggered activation of JNK to suppress HMGB1 release in RAW 264.7 cells

**Fig. 8:**
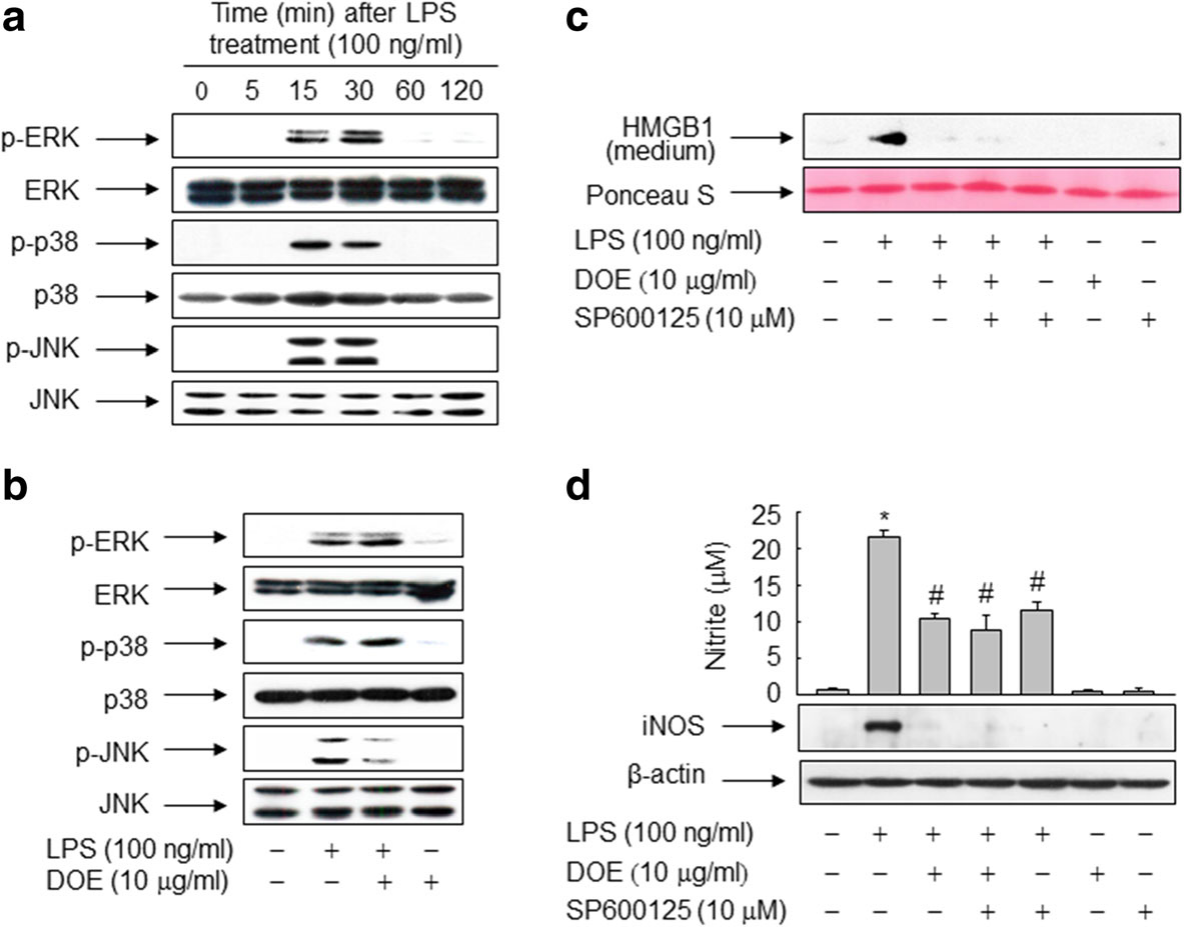
DOE inhibits the LPS-triggered release of HMGB1 by suppressing phosphorylation of JNK via NO in RAW 264.7 cells. **a** Cells were treated with LPS for the indicated amounts of time. **b** Cells were stimulated with vehicle (DMSO) or LPS in the presence or absence of DOE for 30 min. Aliquots of whole-cell lysates were immunoblotted with activation-specific antibodies, and parallel immunoblots were analyzed for total kinase levels. **c** and **d** Cells were pretreated with SP600125 for 1 h and then exposed to vehicle (DMSO) or LPS with or without DOE. Following incubation for 24 h, aliquots of conditioned media or whole-cell lysates were subjected to immunoblot or nitrite analysis to detect HMGB1 (**c**) or iNOS and nitrite (**d**), respectively. The blots are representative of three independent experiments. The results are plotted as the means ± S.E. (*n* = 5). ^*^, *p* < 0.01 vs untreated group; ^#^, *p* < 0.01 vs LPS-treated group

To characterize the signaling pathways involved in DOE-mediated inhibition of HMGB1, we analyzed the involvement of the mitogen-activated protein kinase (MAPK) signaling cascade in LPS-triggered release of HMGB1. In RAW 264.7 cells exposed to LPS, three MAPK cascades were activated soon after LPS treatment (Fig. 8a). Among these, DOE markedly inhibited LPS-induced phosphorylation of JNK, but not the ERK and p38 pathways (Fig. 8b).

To further investigate whether the JNK signaling pathway is involved in LPS-induced HMGB1 release, we examined the effect of a specific inhibitor of JNK on RAW 264.7 cells exposed to LPS. The LPS-induced increase in HMGB1 release was almost completely abolished in the presence of SP600125, an inhibitor of JNK, and the extent of inhibition was similar to that achieved with DOE (Fig. 8c). However, the results obtained upon combined treatment with both DOE and SP600125 were not different from those obtained upon treatment with either alone, indicating that the action of DOE in the inhibition of HMGB1 release is mediated via the JNK signaling pathway. In addition, SP600125 and DOE inhibited NO formation and iNOS expression to a similar extent in LPS-exposed RAW 264.7 cells and there was no additive effect upon combined treatment, suggesting that JNK mediates DOE-mediated inhibition of HMGB1 release via the NO signaling cascade (Fig. 8d).

### DOE attenuates LPS-triggered lethality by inhibiting HMGB1 release

**Fig. 9:**
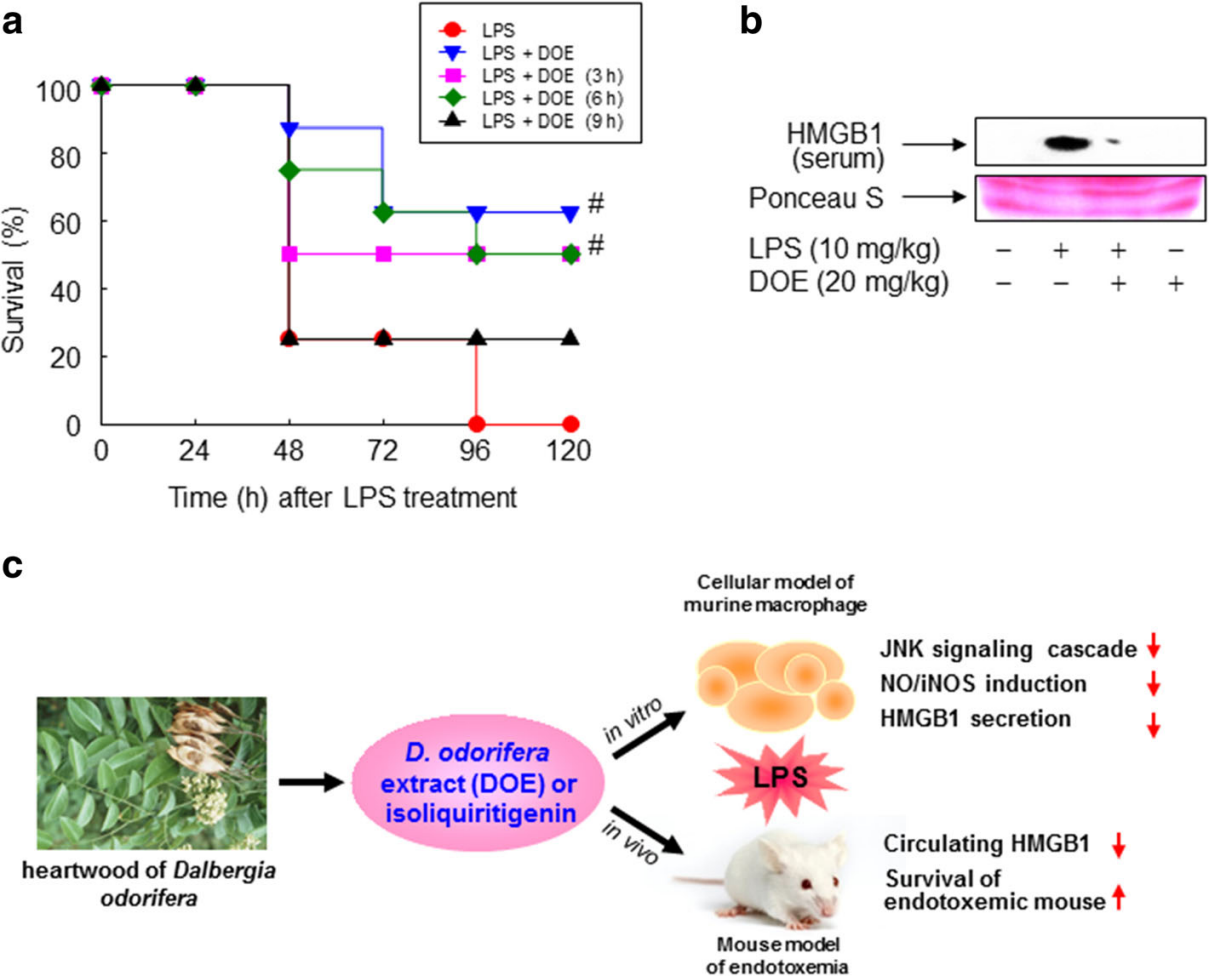
DOE improves the survival rates of mice treated with endotoxin through blockade of HMGB1 release. **a** BALB/c mice (*n* = 8 per group) were treated with LPS (10 mg/kg, i.p.) and then injected with a single dose of DOE (20 mg/kg, i.p.) 0, 3, 6, and 9 h later. Motality was monitored for up to 2 weeks every day. **b** BALB/c mice (*n* = 4 per group) were treated with a single dose of DOE (20 mg/kg, i.p.) with or without a lethal injection of endotoxin (LPS, 10 mg/kg, i.p.). Circulating levels of HMGB1 were detected by immunoblot analysis of sera collected at 20 h post-LPS injection. As a loading control, Ponceau S staining was used. **c** Schematic representation of DOE-mediated inhibition of HMGB1 release. ^#^, *p* < 0.01 vs LPS-treated group

To further elucidate the in vivo relevance of these in vitro observations, the effect of DOE was examined in an animal model of endotoxemia. Injection of endotoxin increased the mortality of mice, whereas simultaneous treatment with DOE and LPS significantly enhanced the survival rates (Fig. 9a). Furthermore, treatment with DOE following LPS treatment also significantly improved the survival rates even up to 6 h post-LPS treatment, indicating that DOE has an extended therapeutic window. Late deaths of DOE-treated animals were not detected during the 2 weeks after LPS injection (data not shown), suggesting that DOE conferred protection of mice against lethal endotoxemia. Because endotoxin mortality is associated with HMGB1 release [11], we investigated the effect of DOE on the level of circulating HMGB1 in blood. The blood level of HMGB1 was markedly increased by LPS injection, whereas the elevated level of HMGB1 in blood was significantly suppressed by administration of DOE with LPS (Fig. 9b). These results suggested that DOE impedes endotoxin mortality in vivo through inhibition of HMGB1 release into the systemic circulation.

## Discussion

Despite recent advances in therapeutics, sepsis remains one of the most lethal incurable diseases. As part of our ongoing efforts to identify natural products with anti-inflammatory activity, we report here that an ethanol extract of *D. odorifera* and its constituent isoliquiritigenin inhibited LPS-triggered release of HMGB1 by blocking NO signaling, which was mediated via suppression of JNK. Furthermore, administration of DOE significantly improved the survival rates in a mouse model of endotoxemia, with a concomitant reduction in the level of circulating HMGB1.

To our knowledge, this is first report demonstrating that DOE inhibits HMGB1 release in murine RAW264.7 macrophages exposed to LPS. The anti-inflammatory effect of DOE may be due to its effects on cellular signaling cascades, as demonstrated in this study. In fact, the neoflavonoid latifolin isolated from a methanol extract of *D. odorifera* attenuates inflammatory responses by inactivating nuclear factor (NF)-κB through nuclear transcription factor-E2-related factor 2 (Nrf-2)-mediated upregulation of HO-1 [22]. In line with the present finding, isoliquiritigenin, another constituent of *D. odorifera*, is a powerful anti-inflammatory molecule that protects against the effects of LPS by inhibiting interleukin-1β, tumor necrosis factor-α, and NO production in RAW 264.7 cells [30]. The anti-inflammatory activity of 4,2′,5′-trihydroxy-4′-methoxychalcone and butein, other components of *D. odorifera*, was also demonstrated in LPS-treated murine macrophages via upregulation of HO-1, which is involved in the resolution of inflammatory conditions [31, 32]. These findings are in line with our present results demonstrating significant DOE-mediated inhibition of HMGB1 release in RAW 264.7 cells stimulated by LPS. DOE and its active components may therefore also exert its action on the cellular response against inflammation by regulating LPS-induced release of HMGB1, which is a late-phase proinflammatory cytokine that plays an important role in the pathogenesis of sepsis [11].

Although three MAPKs are activated by LPS, they were not equally affected by DOE in murine macrophages. In the present study, DOE markedly reduced the effect of LPS on phosphorylation of JNK, but not ERK or p38. Consistent with this observation, the LPS-induced elevation of HMGB1 release was almost completely abolished by SP600125, an inhibitor of JNK. This finding is consistent with a previously reported study in which HMGB1 release was significantly inhibited by intravenous administration of SP600125 in the heart tissue of ischemia-reperfusion model rats [33]. In normal human bronchial epithelial cells, SP600125 also inhibited HMGB1-mediated inflammatory responses through the inhibition of JNK signaling [34]. In contrast with these reports, a recent study demonstrated that another MAPK, p38, is implicated in ketamine-mediated inhibition of HMGB1 release induced by LPS through upregulation of HO-1 [35]. In addition, p38-dependent inhibition of HMGB1 release was also demonstrated in cellular or animal models of severe acute pancreatitis and endotoxemia upon administration of the natural products glycyrrhizin, Dachengqi decoction, and the tetrahydroisoquinoline alkaloid THI-28 [36–38]. Although the MAPK that is involved in the inhibition of HMGB1 release is controversial, the present data clearly demonstrated that JNK participates in DOE-mediated inhibition of HMGB1 release induced by LPS. Accordingly, the identity of the MAPK signaling pathway that is involved in the inhibition of HMGB1 release triggered by inflammatory stimuli may differ according to the nature of the individual compound used.

Among the regulatory mechanisms implicated in HMGB1 secretion, DOE and its constituent isoriquiritigenin inhibit LPS-triggered release of HMGB1 through the NO signaling cascade. The free radical gas NO, a small molecule mediator of inflammation, specifically inhibits HMGB1 release induced by LPS [29]. Post-translational modifications such as acetylation and phosphorylation are the main regulatory mechanism underlying HMGB1 translocation in the cellular responses against bacterial endotoxin or proinflammatory cytokines [39, 40]. Consistent with these findings, our previous study showed that rosiglitazone and GW501516, which are specific activators of PPARγ and δ, respectively, inhibit the LPS-induced release of HMGB1 through an acetylation-dependent manner [13]. Although the functional significance of the inhibition of HMGB1 release has been documented for diverse herbal extracts and components [18–21], the signaling cascades associated with the inhibition of HMGB1 release are now being elucidated. Accordingly, mechanistic studies, including the present data, on the signaling pathway involved in HMGB1 release and the post-translational modifications of HMGB1 itself are important to elucidate the anti-inflammatory actions of DOE via the inhibition of HMGB1 release.

In line with our findings in cultured murine macrophages, administration of DOE significantly attenuated endotoxin-induced lethality in vivo. Upon exposure to the bacterial endotoxin LPS, the level of circulating HMGB1 was reduced in mice treated with DOE, which was accompanied by an improvement in their survival rate, indicating that the effects of DOE on septic shock are HMGB1-dependent. Furthermore, administration of DOE, either together with LPS or after LPS treatment, inhibited LPS-triggered release of HMGB1, suggesting that DOE-mediated regulation of HMGB1 release is effective for both prevention and treatment. The present observation is consistent with previous reports in which blockade of HMGB1 attenuated endotoxin lethality in a mouse model of endotoxemia, even when administered after LPS injection [4, 10, 11]. DOE has also been known to regulate inflammatory reactions by inhibiting nuclear transcription factors, such as NF-κB and Nrf-2, thereby modulating expression of pro- or anti-inflammatory genes [22, 23]. However, little is known about the role of DOE in the regulation of HMGB1 release. Accordingly, the present observations indicate that DOE has anti-inflammatory constituents, which may play a pivotal role in pathological conditions. When seen in this light, inhibition of HMGB1 release in immune cells, which is possibly augmented in inflammatory conditions, may resolve the immune responses in the pathology of endotoxemia. Consequently, inhibitors of HMGB1 release, such as DOE, hold promise as therapeutic interventions in septic shock.

## Conclusion

The present investigation indicates that ethanol extract of *D. odorifera* and its constituent isoliquiritigenin possess the promising anti-inflammatory activities in in vitro and/or in vivo models (Fig. 9c). When seen in this light, inhibition of HMGB1 release in immune cells, which is possibly augmented in inflammatory conditions, may resolve the immune responses in the pathology of endotoxemia. Consequently, inhibitors of HMGB1 release, such as DOE and possibly isoliquiritigenin, hold promise as therapeutic interventions in septic shock.

## Abbreviations

DOE: *D. odorifera* extract
ERK: Extracellular signal-regulated kinase
HMGB1: High mobility group box 1
HO-1: Heme oxygenase 1
iNOS: Inducible NO synthase
JNK: c-Jun N-terminal kinase
LPS: Lipopolysaccharide
MAPK: Mitogen-activated protein kinase
NF-κB: Nuclear factor-κB
NO: Nitric oxide
Nrf-2: Nuclear transcription factor-E2-related factor 2
PPAR: Peroxisome proliferator-activated receptor

## References

[1] Müller S, Ronfani L, Bianchi ME (2004). Regulated expression and subcellular localization of HMGB1, a chromatin protein with a cytokine function. J Intern Med.

[2] Ueda T, Yoshida M (2010). HMGB proteins and transcriptional regulation. Biochim Biophys Acta.

[3] Andersson U, Tracey KJ (2011). HMGB1 is a therapeutic target for sterile inflammation and infection. Annu Rev Immunol.

[4] Wang H, Bloom O, Zhang M, Vishnubhakat JM, Ombrellino M, Che J, Frazier A, Yang H, Ivanova S, Borovikova L, Manogue KR, Faist E, Abraham E, Andersson J, Andersson U, Molina PE, Abumrad NN, Sama A, Tracey KJ (1999). HMG-1 as a late mediator of endotoxin lethality in mice. Science.

[5] Yang H, Ochani M, Li J, Qiang X, Tanovic M, Harris HE, Susarla SM, Ulloa L, Wang H, DiRaimo R, Czura CJ, Wang H, Roth J, Warren HS, Fink MP, Fenton MJ, Andersson U, Tracey KJ (2004). Reversing established sepsis with antagonists of endogenous high- mobility group box 1. Proc Natl Acad Sci U S A.

[6] Sundén-Cullberg J, Norrby-Teglund A, Rouhiainen A, Rauvala H, Herman G, Tracey KJ, Lee ML, Andersson J, Tokics L, Treutiger CJ (2005). Persistent elevation of high mobility group box-1 protein (HMGB1) in patients with severe sepsis and septic shock. Crit Care Med.

[7] Davé SH, Tilstra JS, Matsuoka K, Li F, DeMarco RA, Beer-Stolz D, Sepulveda AR, Fink MP, Lotze MT, Plevy SE (2009). Ethyl pyruvate decreases HMGB1 release and ameliorates murine colitis. J Leukoc Biol.

[8] Harris HE, Andersson U, Pisetsky DS (2012). HMGB1: a multifunctional alarmin driving autoimmune and inflammatory disease. Nat Rev Rheumatol.

[9] Andrassy M, Volz HC, Igwe JC, Funke B, Eichberger SN, Kaya Z, Buss S, Autschbach F, Pleger ST, Lukic IK, Bea F, Hardt SE, Humpert PM, Bianchi ME, Mairbäurl H, Nawroth PP, Remppis A, Katus HA, Bierhaus A (2008). High-mobility group box-1 in ischemia-reperfusion injury of the heart. Circulation.

[10] Ulloa L, Ochani M, Yang H, Tanovic M, Halperin D, Yang R, Czura CJ, Fink MP, Tracey KJ (2002). Ethyl pyruvate prevents lethality in mice with established lethal sepsis and systemic inflammation. Proc Natl Acad Sci U S A.

[11] Wang H, Liao H, Ochani M, Justiniani M, Lin X, Yang L, Al-Abed Y, Wang H, Metz C, Miller EJ, Tracey KJ, Ulloa L (2004). Cholinergic agonists inhibit HMGB1 release and improve survival in experimental sepsis. Nat Med.

[12] Hwang JS, Kang ES, Ham SA, Yoo T, Lee H, Paek KS, Park C, Kim JH, Lim DS, Seo HG (2012). Activation of peroxisome proliferator-activated receptor γ by rosiglitazone inhibits lipopolysaccharide-induced release of high mobility group box 1. Mediat Inflamm.

[13] Hwang JS, Lee WJ, Kang ES, Ham SA, Yoo T, Paek KS, Lim DS, Do JT, Seo HG (2014). Ligand-activated peroxisome proliferator-activated receptor-δ and -γ inhibit lipopolysaccharide-primed release of high mobility group box 1 through upregulation of SIRT1. Cell Death Dis.

[14] Hwang JS, Choi HS, Ham SA, Yoo T, Lee WJ, Paek KS, Seo HG (2015). Deacetylation-mediated interaction of SIRT1-HMGB1 improves survival in a mouse model of endotoxemia. Sci Rep.

[15] Wang H, Li W, Li J, Rendon-Mitchell B, Ochani M, Ashok M, Yang L, Yang H, Tracey KJ, Wang P, Sama AE (2006). The aqueous extract of a popular herbal nutrient supplement, Angelica Sinensis, protects mice against lethal endotoxemia and sepsis. J Nutr.

[16] Zhu S, Li W, Li J, Jundoria A, Sama AE, Wang H (2012). It is not just folklore: the aqueous extract of Mung bean coat is protective against sepsis. Evid Based Complement Alternat Med.

[17] Jun MS, Kim HS, Kim YM, Kim HJ, Park EJ, Lee JH, Lee KR, Kim YS, Chang KC (2012). Ethanol extract of *Prunella vulgaris* Var. Lilacina inhibits HMGB1 release by induction of heme oxygenase-1 in LPS-activated RAW 264.7 cells and CLP-induced septic mice. Phytother Res.

[18] Wu AH, He L, Long W, Zhou Q, Zhu S, Wang P, Fan S, Wang H (2015). Novel mechanisms of herbal therapies for inhibiting HMGB1 secretion or action. Evid Based Complement Alternat Med.

[19] Sakamoto R, Okano M, Takena H, Ohtsuki K (2001). Inhibitory effect of glycyrrhizin on the phosphorylation and DNA-binding abilities of high mobility group proteins 1 and 2 in vitro. Biol Pharm Bull.

[20] Li W, Zhu S, Li J, Assa A, Jundoria A, Xu J, Fan S, Eissa NT, Tracey KJ, Sama AE, Wang H (2011). EGCG stimulates autophagy and reduces cytoplasmic HMGB1 levels in endotoxin-stimulated macrophages. Biochem Pharmacol.

[21] Zhang Y, Li W, Zhu S, Jundoria A, Li J, Yang H, Fan S, Wang P, Tracey KJ, Sama AE, Wang H (2012). Tanshinone IIA sodium sulfonate facilitates endocytic HMGB1 uptake. Biochem Pharmacol.

[22] Lee DS, Kim KS, Ko W, Li B, Keo S, Jeong GS, Oh H, Kim YC (2014). The neoflavonoid latifolin isolated from MeOH extract of Dalbergia Odorifera attenuates inflammatory responses by inhibiting NF-κB activation via Nrf2-mediated heme oxygenase-1 expression. Phytother Res.

[23] Lee DS, Li B, Keo S, Kim KS, Jeong GS, Oh H, Kim YC (2013). Inhibitory effect of 9-hydroxy-6,7-dimethoxydalbergiquinol from Dalbergia Odorifera on the NF-κB-related neuroinflammatory response in lipopolysaccharide-stimulated mouse BV2 microglial cells is mediated by heme oxygenase-1. Int Immunopharmacol.

[24] Goda Y, Kiuchi F, Shibuya M, Sankawa U (1992). Inhibitors of prostaglandin biosynthesis from Dalbergia Odorifera. Chem Pharm Bull (Tokyo).

[25] Chan SC, Chang YS, Wang JP, Chen SC, Kuo SC (1998). Three new flavonoids and antiallergic, anti-inflammatory constituents from the heartwood of Dalbergia Odorifera. Planta Med.

[26] Ham SA, Hwang JS, Kang ES, Yoo T, Lim HH, Lee WJ, Paek KS, Seo HG (2015). Ethanol extract of Dalbergia Odorifera protects skin keratinocytes against ultraviolet B-induced photoaging by suppressing production of reactive oxygen species. Biosci Biotechnol Biochem.

[27] Seo HG, Nishinaka T, Yabe-Nishimura C (2000). Nitric oxide up-regulates aldose reductase expression in rat vascular smooth muscle cells: a potential role for aldose reductase in vascular remodeling. Mol Pharmacol.

[28] Tang D, Kang R, Xiao W, Wang H, Calderwood SK, Xiao X (2007). The anti-inflammatory effects of heat shock protein 72 involve inhibition of high-mobility-group box 1 release and proinflammatory function in macrophages. J Immunol.

[29] Jiang W, Pisetsky DS (2006). The role of IFN-alpha and nitric oxide in the release of HMGB1 by RAW 264.7 cells stimulated with polyinosinic-polycytidylic acid or lipopolysaccharide. J Immunol.

[30] Lee SH, Kim JY, Seo GS, Kim YC, Sohn DH (2009). Isoliquiritigenin, from Dalbergia Odorifera, up-regulates anti-inflammatory heme oxygenase-1 expression in RAW264.7 macrophages. Inflamm Res.

[31] Cheng ZJ, Kuo SC, Chan SC, Ko FN, Teng CM (1998). Antioxidant properties of butein isolated from Dalbergia Odorifera. Biochim Biophys Acta.

[32] Lee DS, Li B, Im NK, Kim YC, Jeong GS (2013). 4,2′,5′-trihydroxy-4′-methoxychalcone from Dalbergia Odorifera exhibits anti-inflammatory properties by inducing heme oxygenase-1 in murine macrophages. Int Immunopharmacol.

[33] Zhai CL, Zhang MQ, Zhang Y, Xu HX, Wang JM, An GP, Wang YY, Li L (2012). Glycyrrhizin protects rat heart against ischemia-reperfusion injury through blockade of HMGB1-dependent phospho-JNK/Bax pathway. Acta Pharmacol Sin.

[34] Wu X, Mi Y, Yang H, Hu A, Zhang Q, Shang C (2013). The activation of HMGB1 as a progression factor on inflammation response in normal human bronchial epithelial cells through RAGE/JNK/NF-κB pathway. Mol Cell Biochem.

[35] Wang F, Meng Y, Zhang Y, Zhao G, Zheng X, Xiao Q, Yu Y (2015). Ketamine reduces lipopolysaccharide-induced high-mobility group box-1 through heme oxygenase-1 and nuclear factor erythroid 2-related factor 2/ p38 mitogen-activated protein kinase. J Surg Res.

[36] Kim YM, Kim HJ, Chang KC (2015). Glycyrrhizin reduces HMGB1 secretion in lipopolysaccharide-activated RAW 264.7 cells and endotoxemic mice by p38/Nrf2-dependent induction of HO-1. Int Immunopharmacol.

[37] Chen Z, Chen Y, Pan L, Li H, Tu J, Liu C, Dai X, Zhang X, Sun G, Feng D (2015). Dachengqi decoction attenuates inflammatory response via inhibiting HMGB1 mediated NF-κB and P38 MAPK signaling pathways in severe acute pancreatitis. Cell Physiol Biochem.

[38] Kim HS, Park EJ, Park SW, Kim HJ, Chang KC (2013). A tetrahydroisoquinoline alkaloid THI-28 reduces LPS-induced HMGB1 and diminishes organ injury in septic mice through p38 and PI3K/Nrf2/HO-1 signals. Int Immunopharmacol.

[39] Bonaldi T, Talamo F, Scaffidi P, Ferrera D, Porto A, Bachi A, Rubartelli A, Agresti A, Bianchi ME (2003). Monocytic cells hyperacetylate chromatin protein HMGB1 to redirect it towards secretion. EMBO J.

[40] Youn JH, Shin JS (2006). Nucleocytoplasmic shuttling of HMGB1 is regulated by phosphorylation that redirects it toward secretion. J Immunol.

